# CRISPR-Cas13: A new technology for the rapid detection of pathogenic microorganisms

**DOI:** 10.3389/fmicb.2022.1011399

**Published:** 2022-10-28

**Authors:** Zhanchao Huang, Jianhua Fang, Min Zhou, Zhenghua Gong, Tianxin Xiang

**Affiliations:** ^1^Medical Center for Major Public Health Events in Jiangxi Province, The First Affiliated Hospital of Nanchang University, Nanchang, China; ^2^Jiangxi Zhongke Yanyuan Biotechnology Co., Ltd., Nanchang, China

**Keywords:** CRISPR, Cas13, rapid detection, pathogenic, microorganisms

## Abstract

Pathogenic microorganisms have major impacts on human lives. Rapid and sensitive diagnostic tools are urgently needed to facilitate the early treatment of microbial infections and the effective control of microbial transmission. CRISPR-Cas13 employs programmable RNA to produce a sensitive and specific method with high base resolution and thus to provide a novel tool for the rapid detection of microorganisms. The review aims to provide insights to spur further development by summarizing the characteristics of effectors of the CRISPR-Cas13 system and by describing the latest research into its application in the rapid detection of pathogenic microorganisms in combination with nucleic acid extraction, isothermal amplification, and product detection.

## Introduction

In 2014, an outbreak of the Ebola virus affected more than 28,000 people and caused at least 11,000 deaths (Fasina et al., 2014; Cenciarelli et al., 2015). In addition, as of September 2021, the cumulative number of confirmed infections with severe acute respiratory syndrome coronavirus 2 (SARS-CoV-2) globally has exceeded 225 million, and the associated disease, COVID-19, has caused more than 4.6 million deaths.^1^ The curbing of the spread of these two viruses has been challenging, especially as both viruses have been shown to be transmittable *via* aerosols (van Doremalen et al., 2020; Downs et al., 2021). The transmission of bacteria, viruses, and other pathogenic microorganisms can occur in multiple ways, such as contact with surfaces or the air or even through the food chain and water reservoirs (Szewzyk et al., 2000). The resulting rapid transmission can affect a wide range of hosts, leading to local or even global outbreaks. These outbreaks can overwhelm control efforts, significantly endangering human health and resulting in substantial economic losses and potential social instability.

The rapid and accurate diagnosis of these infections, as well as screening of populations for the pathogens, is key to limiting such outbreaks and to the implementation of early life-saving treatments (Qiu et al., 2020). Conventional methods of identification of pathogenic microorganisms involve the examination of morphology and the identification of molecular characteristics or the presence of biomarkers. However, these methods tend to rely on complex and time-consuming procedures, and some microorganisms with complex growth properties are undetectable, making it difficult to meet clinical needs for methods to rapidly detect pathogenic microorganisms (Craw and Balachandran, 2012; Hassan et al., 2018).

Polymerase chain reaction (PCR), which can produce billions of copies of even a single DNA fragment based on specific nucleic acid sequences, is the gold standard for the detection of nucleic acids of pathogenic microorganisms. Although PCR has proven indispensable as a method for detecting pathogenic microorganisms, sample processing presented multiple challenges during the SARS-CoV-2 epidemic. These challenges included long sample processing times and issues with transporting samples for the mass screening of populations, and they are likely to limit the speed with which SARS-CoV-2 and other pathogens can be detected.

Conversely, the point-of-care test (POCT) is a means of instant detection that may be useful for the rapid detection of viral or bacterial pathogens during epidemic outbreaks. The POCT has multiple positive characteristics, including the possibility for rapid, automated processing and high precision and accuracy (Fernandes et al., 2022). In addition, unlike PCR, this detection method is not dependent on laboratory conditions and thus has the potential for large-scale detection. Thus, POCT has important advantages for rapid detection during large-scale epidemics.

## Summary of the CRISPR-Cas system

A relatively new technology based on clustered regularly interspaced short palindromic repeats (CRISPR) and CRISPR-associated sequences (Cas; Barrangou, 2013) represents a potential improvement to the potential of PCR. The CRISPR-Cas system has been used most famously for gene editing, but it has also been shown to be a powerful and accurate diagnostic tool (Huang et al., 2018; Curti et al., 2020). CRISPR-Cas systems, including CRISPR-Cas9 (Park et al., 2017), CRISPR-Cas12a (Zetsche et al., 2015), and CRISPR-Cas13a (Shmakov et al., 2015), have been discovered to recognize and cleave specific DNA and RNA fragments. In particular, CRISPR-Cas12a and CRISPR-Cas13a can cleave DNA and RNA adjacent to target sequences, a feature that can be used to detect pathogenic microorganisms. Even though these systems are relatively new, current diagnostic tests based on CRISPR-Cas systems have proven to be capable of detecting the presence of the microbial genome at any stage of infection, even in the early stages when PCR results are usually negative.

The CRISPR-Cas system functions as an adaptive immune process that allows bacteria and archaea to defend themselves against invasive viruses and plasmids. The CRISPR-Cas system is composed of a leader sequence (LS), a CRISPR locus and a CRISPR-associated gene (Cas gene; Santiago-Frangos et al., 2021). The CRISPR locus is composed of repeat sequences and spacer sequences of similar length. The sequence of the CRISPR array has double symmetry so that it can form a hairpin structure, which is the binding region of the Cas protein (Kunin et al., 2007). When exogenous DNA enters bacteria or archaea that employ the CRISPR-Cas system, the CRISPR-associated protein complex in the host binds to protospacer adjacent motifs (PAM) of the exogenous DNA, and a DNA sequence adjacent to the PAM is used as a candidate protospacer sequence. Then, under the action of related proteins, the protospacer sequence is integrated between the LS and the first repeat sequence to form a new spacer sequence (Zhang and An, 2022). The sequence information from the exogenous DNA that is stored between the repeats then serves as an immunological memory (Bhaya et al., 2011).

CRISPR-Cas systems are divided into two classes based on whether the protein component of the crRNA-Cas effector complex is a complex of several protein subunits (class 1) or a single multidomain protein (class 2; Chaudhuri et al., 2022). The class 1 CRISPR-Cas systems consist of types I, III, and IV. The Cas3 gene plays a major role in the type I CRISPR-Cas system. The Cas3 protein encoded by it has both helicase and DNase activity, and it is the main enzyme deployed during the interference stage. Type III systems employ polymerase and repeat-associated mysterious protein (RAMP) molecules. These systems that recognize and target RNA include the type III-A and type III-B systems and other subtypes. Both have also been shown to stimulate non-specific, collateral RNA degradation and to have single-stranded DNase activity (Makarova et al., 2011; Kolesnik et al., 2021; van Beljouw et al., 2022).

Class 2 consists of types II, V, and VI. The type II CRISPR-Cas system is the most extensively studied of the systems, and it contains three subtypes, including the representative Cas9 protein (Chylinski et al., 2014). The type V CRISPR-Cas system includes 11 subtypes that cleave target DNAs. A characteristic Cas protein among type V systems is Cas12 (Zetsche et al., 2015; Harrington et al., 2018; Makarova et al., 2020). The type VI CRISPR-Cas system is defined by the Cas13 nuclease, and it has four subtypes (Anantharaman et al., 2013). Thus far, four Cas13 families have been identified, including Cas13a (c2c2; Abudayyeh et al., 2016), Cas13b (c2c6), Cas13c (Smargon et al., 2017), and Cas13d (c2c7; Shmakov et al., 2017; Konermann et al., 2018). Due to its targeted cleavage of RNA, it is widely used in virus detection.

The Specific High-Sensitivity Enzymatic Reporter UnLOCKing (SHERLOCK) detection platform is based on the CRISPR-Cas13a system and is guided by crRNA-targeted ssRNA. It cleaves RNA adjacent to a targeted region. This activity potentially provides a defense against RNA viruses that infect eukaryotic cells and against the RNA intermediates of DNA viruses (Mahas et al., 2018), but it also permits fluorescent-based viral detection. Notably, using the CRISPR system to target RNA genomes would not cause permanent changes to the host genome (Mahas and Mahfouz, 2018).

Another platform based on the CRISPR-Cas9 system (Park et al., 2017) enhances detection accuracy through recognition of PAM sequences in the target DNA (Kim et al., 2017). However, CRISPR-Cas9 is limited by the need to carefully select of the target nucleic acid region and its possible off-target effects. In contrast to the disadvantages of the Cas9 system, the CRISPR-Cas13 system is able to target any possible sequence based only on a simple protospacer flanking site (PFS) that consists only of a base other than guanine. Some Cas13 homologous genes, such as LwaCas13a, do not even require a PFS (Gootenberg et al., 2017), thus suggesting more extensive possible applications of CRISPR-Cas13 (Table 1).

**Table 1:** The diversity and characteristics of the CRISPR-Cas system.

Type	Class	Effector Nuclease	Subtypes
I	1	Cas1, Cas2, Cas3, Cas4, Cas5, Cas6 (Cas6, Cas6e, Cas6f), Cas7, Cas8 (Cas8a1, Cas8a2, Cas8b, Cas8c), Cas10d	I-A, B, C, D (Csc), E (Cse), F (Cys), G, U
II	2	Cas1, Cas2, Cas4, Cas9	II-A, B, C, II-Cvariant
III	1	Cas1, Cas2, Cas5, Cas6, Cas7, Cas10, Cas11	III-A (Csm), B (Cmr), C, D, III-B variant
IV	1	Cas5, Cas6-like, Cas7, Cas8-like, Cas11	IV, IV-variant
V	2	Cas1, Cas2, Cas4, Cas12, Cas14	V-A, B, C, D, E, U(1–5), F
VI	2	Cas1, Cas2, Cas13	VI-A, B1, B2, C,

### Composition and mechanism of the CRISPR-Cas13 system

#### Cas13 protein

The CRISPR-Cas13 system has two components: the Cas13 protein effector and a CRISPR RNA (crRNA) of 64 to 66 nucleotides (Koonin et al., 2017; Granados-Riveron and Aquino-Jarquin, 2021). In 2016, Wang et al. (Liu et al., 2017b) analyzed the crystal structure of the Cas13a protein and the secondary structure of crRNA. They found that the Cas13a protein extracted from the Gram-negative bacterium *Leptotrichia shahii* (LshCas13a) catalyzes two reactions to both processes mature crRNA and cleave the target RNA. The Cas13a protein is composed of crRNA-recognition (REC) lobe and nuclease (NUC) domain. The REC lobe contains an N-terminal domain (NTD) and a domain called helix-1 or Helical-1. The NTD is a non-conserved region of Cas13a that consists of a larger subdomain containing an ordered fragment composed of seven α helices and a disordered fragment, and a smaller subdomain containing three α helices, a β-hairpin and a β-sheet.

The helix-1 domain contains seven α helices, forming a V-shaped structure. The surface of the helix-1 domain that faces the NTD domain is positively charged, thus forming the crRNA-binding channel (Liu et al., 2017b). The NUC lobe contains two conserved higher eukaryotes and prokaryotes nucleotide-binding (HEPN) domains (HEPN1 and HEPN2), a linker that connects the two HEPN domains and a helix-2 domain. The two enzymatic activities of Cas13a are attributed to the helix-1 and HEPN domains.

**Figure 1:**
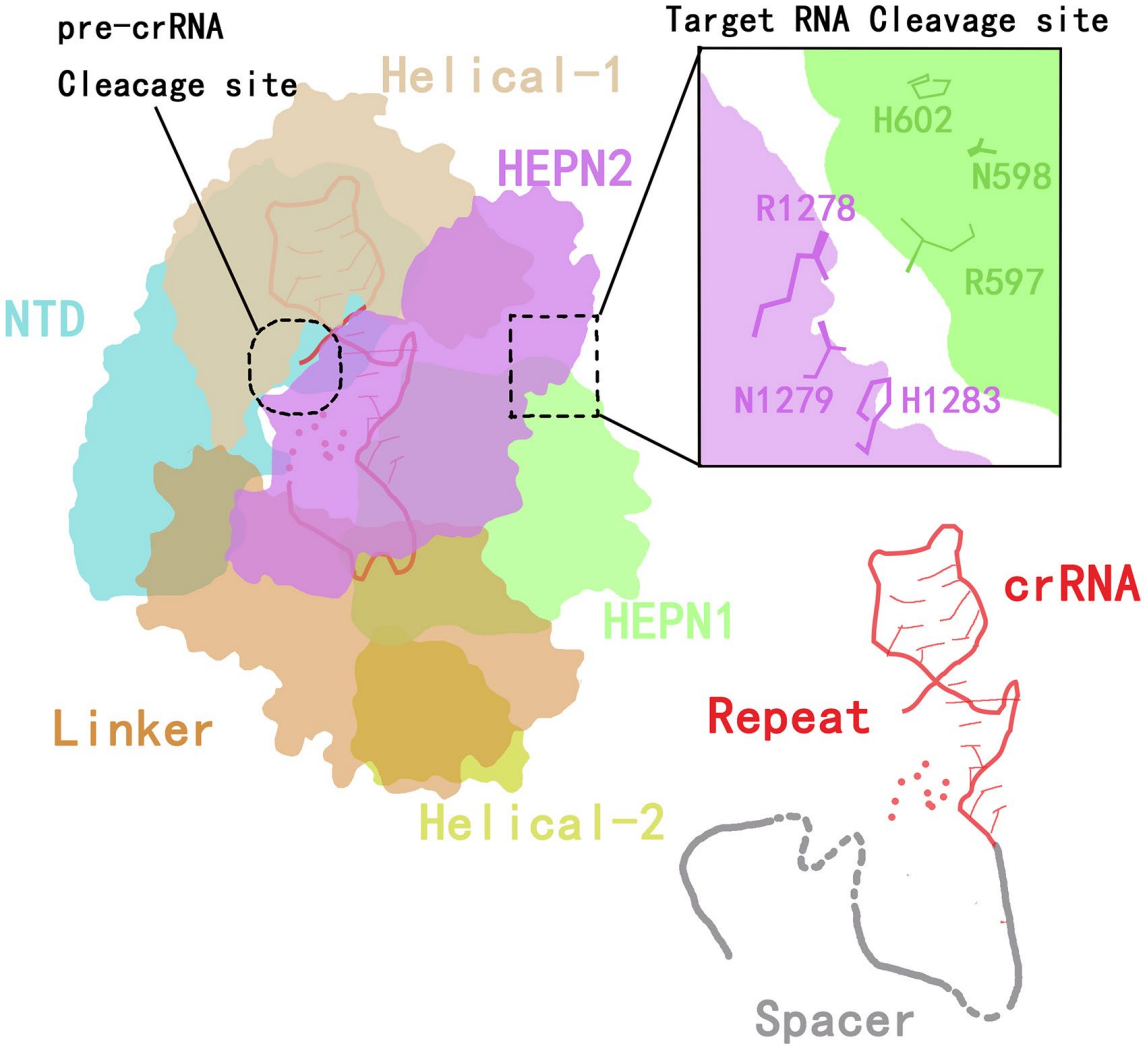
The crystal structure of Cas13a in the crRNA complex.

The HEPN1 domain is further divided into two subdomains by the helix-2 domain (yellow in Figure 1). The HEPN1-I subdomain consists of four α helices and a short β-hairpin, while the HEPN1-II subdomain consists of three α helices. The structure of the HEPN2 domain consists of seven α helices and a double-stranded β-sheet directly. The helix-2 domain is located between the two HEPN1 subdomains, and it consists of eight α helices in a bean shape (Liu et al., 2017b). The assembling of Cas13a and crRNA forms a Cas13a/crRNA complex. Under the guidance of crRNA, Cas13a targets the corresponding RNA, thus inducing the immunity of prokaryotes against RNA viruses (Abudayyeh et al., 2016; Mahas et al., 2019).

#### crRNA structure

The structure of the crRNA consists of a repeat stem-loop region (called the 5′-handle) that protects this region from being cleaved during the cleavage of the target RNA by Cas13a (Yang and Chen, 2017), and a spacer sequence (guide RNA, gRNA) that mediates target recognition by RNA–RNA hybridization. The stem-loop contains a stem formed by five base pairings, one loop with nine bases, adjacent motifs at both ends, and two single-stranded bases at the base. The stem-loop is located between NDT and helix-1; this localization is stabilized by hydrogen bonding and aromatic stacking within the stem-loop structure and by charge interactions between crRNA and NTD and between the helix-1 and HEPN2 domains (Liu et al., 2017b).

In addition to the formation of a stem-loop secondary structure, the repeat region of crRNA can be slightly twisted to form a helical structure that is mainly maintained by the formation of extensive hydrogen bonds between the HEPN2 domain and the crRNA backbone (Liu et al., 2017b). By mutating stem nucleotides, the Cas13a protein can be induced to recognize the stem-loop structure of the crRNA in a sequence-specific manner, which is essential for its nuclease cleavage activity (Liu et al., 2017b).

In the guide region of the crRNA, one to four nucleotides at the 5′-end are bound to the crevice formed by the HEPN1 and helix-2 domains, and four or five nucleotides are responsible for forming a U-turn. The remaining three or four nucleotides are bound to the linker and the groove formed by the HEPN2 domain. Therefore, a spacer of eight or nine nucleotides at the 5′-end is buried in the Cas13 protein, and the central region and 3′-end are exposed to the solvent to recognize the target RNA (Liu et al., 2017b). Wessels et al. (2020) developed a computational model to identify the optimal gRNA. By measuring the activities of 24,460 gRNAs and detecting mismatches between gRNAs and the target sequence, they found that the characteristics of crRNA and the environment of the target RNA are key limits to the cleavage efficiency of the Cas protein (Figure 1).

#### The mechanism of cleavage by the CRISPR-Cas13 system

**Figure 2:**
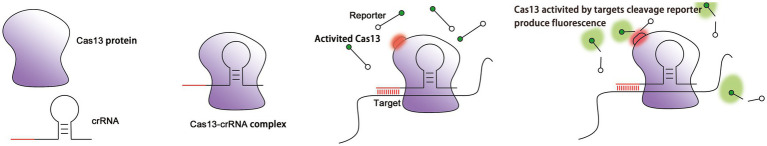
Cleavage of target RNA by the Cas13 protein as guided by crRNA. **(A)** The Cas13 protein localizes the target RNA under the guidance of crRNA. **(B)** Cas13 is activated after crRNA finds and binds to the target RNA (red circle: the structure of HEPN subunit of Cas13 protein changes). **(C)** Activated Cas13 cleaves the target RNA. **(D)** Activated Cas13 not only cleaves the target RNA, but also randomly cleaves other nearby RNAs in a collateral effect. This effect can be detected *via* reporter RNAs with fluorescent and quenching groups at both ends (green: fluorescent group).

The maturation of crRNA is not necessary for the activity of Cas13, and even unprocessed pre-crRNA is sufficient to recognize target RNAs (East-Seletsky et al., 2017). The binding between crRNA and a target RNA triggers a conformational change in the ribonucleoprotein complex (RNP). The close contact between two HEPN domains contributes to the formation of a catalytic site (Liu et al., 2017a). Due to the distance between the catalytic site and the crRNA-RNA duplex, the binding of crRNA is expected not only to lead to cleavage of the target RNA, but also to the cleavage of other ssRNAs that surround the RNP complex, including, potentially, host RNA (Figure 2). Off-target cleavage of endogenous RNA is known as collateral damage or collateral cleavage (Abudayyeh et al., 2016; Smargon et al., 2017; Yan et al., 2018). The sequence-specific activation of non-specific RNA cleavage is speculated to enhance the prevention of phage replication *via* the bulk elimination of all RNA within a cell, or it may be involved in the protection of neighboring cells *via* the inducing of cell dormancy or death (Yan et al., 2018). Therefore, the CRISPR-Cas13 system is able to simultaneously cleave target RNA and non-target RNA, and this propensity has been widely applied to RNA knockout strategies, disease treatment, interference with viral infection, screening of loss of function mutants, molecular detection of biological agents, and CRISPR-based antimicrobials (Anantharaman et al., 2013; Fonfara et al., 2016; Mahas and Mahfouz, 2018).

To expand the use of Cas13 in RNA research, catalytically inactive Cas13 (dCas13) has been created by mutating an arginine in the HEPN domain that is responsible for RNA cleavage. Mutant dCas13 binds stably to target RNA and provides a platform for tracking transcripts in living cells through the binding of fluorescent proteins or enzymes (Abudayyeh et al., 2017; Palaz et al., 2021; Figure 2).

## A rapid detection system based on CRISPR-Cas13

A system based on CRISPR-Cas13 that rapidly detects molecular signatures of specific microbes typically utilizes three main steps: nucleic acid extraction, isothermal amplification, and product detection.

### Nucleic acid extraction

The effective extraction of nucleic acids from biological samples is a necessary step for the accurate diagnosis of microbial infections. Despite the rapid development of nucleic acid extraction technologies, multiple challenges remain. Improvements that need to occur include simplification of procedures, enhancement of the sensitivity of extraction of rare viral nucleic acids from abundant genomic DNA and total RNA, broadening the ability to process various sample types, and the elimination of inhibitors of PCR. These improvements would eliminate important confounding factors that significantly influence the results of subsequent amplification and detection (Nargessi and Ou, 2010). The stability of RNA is another challenge for the rapid detection of viruses. Considering the single-stranded structure of RNA, if the samples cannot be processed quickly after sample collection, external factors such as nucleases will cause the degradation of RNA and make it impossible to detect pathogens. Simple, fast, low-cost, and flexible extraction and purification systems are especially critical for laboratories in resource-poor countries and remote areas (Nargessi and Ou, 2010).

The main types of nucleic acids to be extracted include genomic RNA, genomic DNA, miRNA and plasmid DNA. Three steps are necessary for the extraction of these nucleic acids: cell lysis, nucleoprotein disassociation, and nucleic acid purification.

Common cell lysis methods include physical, chemical, and biological methods. Physical methods include boiling (Zhu et al., 2006; Shin et al., 2021), the use of beads and mechanical agitation (bead beating), ultrasonic vibration, grinding in liquid nitrogen, and freeze–thaw cycles. Chemicals methods use cetyltrimethylammonium bromide (CTAB; Ali et al., 2017), phenol (Cheng and Jiang, 2006), formamide, guanidine hydrochloride, and organic solvents (Sambrook and Russell, 2006), lithium chloride and urea (Tenniswood and Simpson, 1982), sodium dodecyl sulfate (Notomi et al., 2000), alkali (Lahijani et al., 1996; Qin et al., 2019), or a mixture of guanidinium thiocyanate and phenol mixture (TRIzol; Van Ness et al., 2003) to lyse the cell. Then, organic solvents (Vincent et al., 2004), alcohol adsorption materials (Zhang et al., 2014; Li et al., 2019), concentrated salt, density gradient centrifugation, or other methods can be used to purify the nucleic acids. Biological methods use enzymes, including lysozyme and proteinase K, to digest cellular debris to liberate nucleic acids.

Notably, most of these methods rely on centrifugation, which is a resource- and time-intensive procedure that may not be compatible with rapid deployment in low-income or rural areas. In 2018, Zhang et al. (Myhrvold et al., 2018) proposed a novel extraction method called heating unextracted diagnostic samples to obliterate nucleases (HUDSON), which can be used in the identification of pathogenic microorganisms with a high sensitivity. Briefly, samples are first incubated with ethylenediaminetetraacetic acid (EDTA) and the reducing agent tris(2-carboxyethyl) phosphine (TCEP) at 37 to 50°C for a brief 5 to 20 min to dissolve biological particles and eliminate nuclease activity, and then they are reacted at 64 to 95°C for 5 min to release nucleic acids *via* destruction of the bacterial or viral shell. The development of HUDSON is an important breakthrough that greatly shortens the processing time. Nevertheless, it has several limitations, including the need for a heating step and a low sensitivity in the subsequent detection of microbes in serum and urine samples (Myhrvold et al., 2018; Li et al., 2019).

High-quality nucleic acid extraction is necessary for the success of the following procedures and analysis of microorganisms. To make nucleic acid extraction simpler and more robust, a novel room temperature method with simple operation, rapid extraction, and cheap reagents and instruments is urgently needed. The design of a sample pretreatment strategy that is optimally adaptable to CRISPR-based systems is also an area of ongoing investigation.

### Nucleic acid amplification and gene editing technology

Nucleic acid amplification aims to exponentially amplify a target sequence, thus widening the difference in intensity between the detected signal and background signal, allowing detection and reducing resulting error. Since the initial application of PCR in 1983, nucleic acid amplification has been widely applied to life science research (Li et al., 2018), and it has become the gold standard for nucleic acid detection (Saiki et al., 1985; Hillemann et al., 2011).

Isothermal amplification is currently the most popular method for the rapid detection of nucleic acids. This method features high accuracy and applicability for on-site detection. Conventional PCR requires three steps, denaturation, annealing, and extension, and it is thus dependent on an expensive temperature-controlled instrument that limits its application to laboratory environments (Li et al., 2018). Constant temperature amplification, on the other hand, does not require a denaturation process, which accelerates the amplification speed and shortens the reaction time (Zhao et al., 2015; Bodulev and Sakharov, 2020). In addition, some isothermal amplifications realize the goal of portable rapid nucleic acid detection, because they do not require a temperature control instrument (Zhao et al., 2015). Isothermal amplification can better meet the needs of rapid and convenient nucleic acid detection, and has a great potential for on-site, point-of-care, and *in situ* assays (Reid et al., 2018).

Presently, additional commonly used nucleic acid amplification technologies include loop-mediated isothermal amplification (LAMP; Notomi et al., 2000), recombinase polymerase amplification (RPA; Piepenburg et al., 2006), isothermal exponential amplification reaction (EXPAR; Van Ness et al., 2003), helicase-dependent amplification (HDA; Vincent et al., 2004), strand displacement amplification (SDA; Zhang et al., 2014), nucleic acid sequence-based amplification (NASBA; Compton, 1991), and rolling circle amplification (RCA; Li et al., 2009; Table 2). Notably, nucleic acid amplification is frequently combined with Cas13 collateral cleavage technologies to reduce the lower limits of detection.

**Table 2:** Advantages and disadvantages of various nucleic acid amplification methods.

Nucleic acid amplification	Temperature (°C)	Time (h)	Advantages	Disadvantages
Conventional PCR	55–95°C	1–2 h	Gold standard; long-term experience in multiple applications	Long reaction time; end-point detection only; expensive instruments for thermal cycling; frequent product contamination
qPCR	55–95°C	2 h	Real-time amplification and quantitative detection; low incidence of cross-contamination; high sensitivity; high throughput	Long reaction time; expensive instruments needed for thermal cycling
LAMP (Notomi et al., 2000)	60–65°C	0.5-1 h	High amplification efficiency; rapid detection	Requirement for complicated primer design; only useful for short sequences; amplified products can only be quantified, not used for further processing; high sensitivity increases false positive results
RPA (Piepenburg et al., 2006)	37–42°C	20 min	Simple primers; rapid detection; high sensitivity and specificity; easy reagent storage	Complicated and expensive reaction components involving three enzymes; long primers are not suitable for the amplification of short targets
EXPAR (Van Ness et al., 2003)	60°C	>0.5 h	Diverse reactions for different amplification requirements; high sensitivity and specificity; rapid detection	Complicated reaction mechanism and easily restricted by the template, leading to linear amplification rather than exponential amplification
HDA (Vincent et al., 2004)	37–65°C	0.5-2 h	Simple primer design	Unavailable for long sequence amplification
SDA (Zhang et al., 2014)	37–40°C	2 h	High amplification efficiency	Uniform products with drag bands in electrophoresis that cannot be used for sequencing or cloning; unavailable for long sequence amplification; requirement for special detection instrument
NASBA (Compton, 1991)	About 41°C	1.5–2 h	T7 promoter sequence in the primer ensures high specificity and sensitivity in detecting targets with DNA contamination; the combination of transcription and amplification shortens the reaction time	Complicated reaction components involving three enzymes; high cost; mainly used for amplifying RNAs and not suitable for detecting DNA viruses
RCA (Li et al., 2009)		1 h	High sensitivity; high sequence specificity; high throughput	High cost to synthesize 100-bp long padlock probe; possibility of background signals if an unlooped padlock probe does not bind to the template

#### LAMP and CRISPR-Cas13

LAMP is one of the most widely utilized isothermal amplification methods. It efficiently (0.5–1 h) amplifies target DNA at a constant temperature (60–65°C) using the *Bst* DNA polymerase, which has strand displacement activity. The process requires the synthesizing of four specific primers targeting six regions of the target gene (Notomi et al., 2000). However, the use of a long extension time tends to result in the amplification of non-template sequences by the DNA polymerase; therefore, the target sequence should not exceed 300 bp (Notomi et al., 2000; Silva et al., 2019). In addition, LAMP can generate uneven structures within the same template, and the amplification product cannot, then, be used for cloning or sequencing, but only for quantification (Notomi et al., 2000; Silva et al., 2019). LAMP synthesizes a large amount of DNA in a short period of time, resulting in the production of a pyrophosphate precipitate, making the reaction solution turbid (Mori et al., 2001). Therefore, the turbidity of the reaction mixture is an indicator of sequence amplification (Hara-Kudo et al., 2007; Yamazaki et al., 2008), although aerosol pollution can cause false positive results due to the high sensitivity of the procedure.

**Figure 3:**
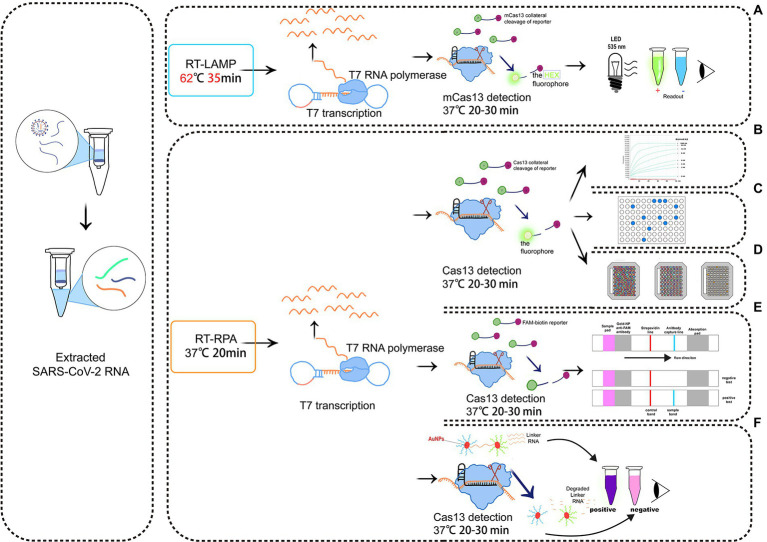
Schematic diagram of a detection system based on Cas13. **(A)** RT-LAMP with the mcas13 system; **(B)** the SHERLOCK system; **(C)** a system consisting of SHERLOCK and ddcas13a; **(D)** a carmen-cas13 (gene chip detection) system; **(E)** a system consisting of SHERLOCK and LFA; **(F)** a system utilizing both SHERLOCK and AuNPs.

Recently, Mahas et al. (2021) established a reverse-transcriptase (RT)-LAMP mCas13 system to detect SARS-CoV-2 from throat swab samples of COVID-19 patients (Figure 3A). The RT-LAMP-amplified product targets crRNA and activates T7 RNA polymerase, which subsequently activates the cleavage of HEX fluorophore-labeled RNA reporters by mCas13. The released fluorescent signals thus can be easily quantified (Mahas et al., 2021). To test the detection specificity of this RT-LAMP Cas13 system, samples are simultaneously analyzed to detect multiple common viruses, either related to COVID-19 or not, including SARS-CoV-1, Middle East respiratory syndrome virus, human coronavirus NL63, human coronavirus OC43, human coronavirus 229E, H1N1 influenza, tobacco mosaic virus, and turnip mosaic virus. When only the background signal is detected, the reliability of the RT-LAMP/mCas13 result is confirmed (Mahas et al., 2021). In addition to COVID-19, LAMP has been used to detect other pathogens, including *Escherichia coli* (Hara-Kudo et al., 2007), *Campylobacter jejuni* (Yamazaki et al., 2008), *Staphylococcus aureus* (Goto et al., 2007), *Salmonella* (Hara-Kudo et al., 2005), *Helicobacter pylori* (Minami et al., 2006), *Gagella* (Minami et al., 2006), and *Vibrio cholerae* (Yamazaki et al., 2008).

#### RPA and CRISPR-Cas13

The procedure of RPA requires mild reaction conditions, from 37 to 42°C, and it amplifies nucleic acids within 20 min (Piepenburg et al., 2006). Only three proteins, typically in the form of a freeze-dried powder, are needed. These proteins are a recombinase enzymes that can act on single-stranded nucleic acids, a single-stranded DNA (ssDNA)-binding protein, and a strand-displacing polymerase. The sensitivity of RPA is comparable to that of LAMP, and, considering the mild conditions and the simplicity of the required reagents, RPA is a promising on-site rapid detection platform. Based on the high sensitivity and specificity of RPA, multi-RT-RPA has been performed in a single tube containing specific primers targeting up to five viruses, with the aim of detecting crRNAs corresponding to different amplification products (Piepenburg et al., 2006; Jiao et al., 2021).

Zhang et al. (Gootenberg et al., 2017) created the SHERLOCK system in 2017. It involves RPA amplification, T7 transcription, and Characteristics of CRISPR-Cas13 collateral cleavage and is able to identify single base differences with a very high sensitivity at the attomolar level (Figure 3B). The SHERLOCK system has been used to specifically detect the Zika virus (ZIKV), Dengue virus (DENV), and pathogenic bacteria and has been used for human genotyping and the identification of free cancerous DNA. Following amplification of the target gene by RPA or of a target RNA by RT-RPA, Cas13a localizes to and cleaves the target RNA under the guidance of the amplified crRNA. Meanwhile, the collateral effect is activated during the detection process. Dinucleotide motifs are cleaved to release fluorescent signals, which are quantified to achieve the detection of the target gene. This application is limited to the detection of a single pathogen at a time.

In 2018, a multiplexed nucleic acid detection platform was developed based on the SHERLOCK system, the quantitative detection limit of which was as low as 2 aM (Gootenberg et al., 2018). Owing to the cleavage preferences of Cas proteins, cleavage of dinucleotides with different fluorescent groups in the same tube by this novel multiplexed platform is able to simultaneously detect ZIKV and DENV, as well as mutations in body fluid biopsy samples. Notably, it is limited by the number of Cas proteins, and the detection results may be influenced by the cross-cleavage of proteins and reporter genes (Yin et al., 2021). Microbial targets at high concentrations cannot be quantified by this method due to the high sensitivity and limited number of reporter molecules (Li et al., 2019). Zhang et al. (Myhrvold et al., 2018) then proposed a novel nucleic acid preparation method, namely HUDSON. Combined with SHERLOCK, HUDSON rapidly detects ZIKV within 90 min using a color test strip, which sensitively detects the virus in whole blood, serum, saliva, and urine samples at sensitivities of 90 aM, 90 aM, 0.9 aM, and 20 aM, respectively. Because it is independent of complex instrumentation, the development of HUDSON further promotes the application of rapid on-site virus detection.

In 2019, Zhang et al. (Freije et al., 2019) again developed a powerful, rapid, and programmable diagnostic and antiviral system to detect RNA viruses, namely Cas13-assisted restriction of viral expression and readout (CARVER). The system is expected to be applicable to the diagnosis and treatment of viral infections, including those caused by newly emerged viruses. They initially screened a series of RNA viruses and searched for viral sequences that Cas13 protein can effectively target, is not easily mutated, and is most likely to disable the virus after being cut off. In addition, thousands of sites in hundreds of viruses were determined to be effective targets for Cas13 through computational analyses. In terms of an antiviral effect, experimental data showed that appropriately targeted Cas13 enzyme drastically reduces the level of lymphocytic choriomeningitis virus (LCMV), influenza A virus (IAV), and vesicular stomatitis virus (VSV) in cells.

To simplify sample processing and the overall procedure, Arizti-Sanz et al. (2020) in 2020 created the diagnostic platform SHINE based on SHERLOCK to detect COVID-19. Incorporating the single-step SHERLOCK, SHINE combines RT-RPA (step 1) and T7 transcription and Cas13-based collateral cleavage (step 2) into a single-step reaction, which reduces the possibility of contamination and shortens the reaction time to 50 min. In addition, the test results can be displayed through fluorescence or a nucleic acid strip, which can be automatically analyzed in a mobile phone application, allowing rapid dissemination of results to users. Owing to the high sensitivity (90%) and specificity (100%), the diagnostic platform SHINE is able to be applied in hospitals and laboratories (Arizti-Sanz et al., 2020).

In 2020, Ackerman et al. (2020) developed the combinatorial arrayed reactions for multiplexed evaluation of nucleic acids (CARMEN) with the Cas13 system (CARMEN-Cas13), which can identify 169 human-related viruses and at least 10 published genome sequences at the same time, and, with the addition of a new crRNA, it can quickly detect COVID-19.

The combination of RT-RPA and the CRISPR-Cas system has been shown to detect COVID-19 with a high sensitivity; it was able to provide positive detection in samples containing as few as 42 RNA copies (Patchsung et al., 2020). Fozouni et al. (2021) created a non-amplification CRISPR-Cas13a system to directly quantify RNAs in COVID-19 samples. It targets multiple sites in viral RNA under the guidance of crRNA, with a sensitivity of about 100 copies/μl. Some other viruses have been detected using the CRISPR-Cas13a-based systems, including canine parvovirus (Khan et al., 2019), avian influenza virus (Liu et al., 2019), Ebola and Lassa viruses (Qin et al., 2019; Barnes et al., 2020), porcine reproductive and respiratory syndrome virus (PRRSV; Chang et al., 2020), and hepatitis B virus (HBV; Wang et al., 2021).

The sensitivity, specificity, efficacy, and convenience of detecting target genes are significantly improved by combining isothermal amplification and CRISPR-Cas13 collateral cleavage technology (Table 3), which also highlights the potential to rapidly and quantitatively detect multiple RNA viruses (Hadidi, 2019). Therefore, this combination has vital clinical significance and deserves to be further analyzed. Nevertheless, the CRISPR-Cas13 system is only effective for known viruses. To diagnose newly emerged viruses, next-generation sequencing (NGS) is needed to identify the genome sequence, but this process is time-consuming (Safari et al., 2021).

**Table 3:** Comparison of the pathogenic microorganism detection systems.

Detection system	Principle	Comparison with the current method	Application
Conventional microorganism detection methods (Craw and Balachandran, 2012; Hassan et al., 2018)	Microorganisms are isolated, cultured, purified, and identified according to phenotypic characteristics.	Complicated and time-consuming procedures; Loss of optimal treatment window; Some microorganisms cannot be cultured.	Gradually being replaced.
PCR	Amplified using the primers specific to the genome sequence, and identified.	Time-consuming; Single detection; Dependent on precise temperature control instrument, professional staff, and laboratories to reduce the occurrence of errors.	Wide range of applications; The gold standard for rapid detection.
SHERLOCK (Gootenberg et al., 2017)	Combination of RPA, T7 transcription, and Cas13 for amplifying the target gene and activating *cis* and *trans* cleavage, thus releasing fluorescent signals.	Only one pathogenic microorganism can be detected at a time.	Available to detect pathogenic microorganisms, drug-resistance genes, and cancer cells.
SHERLOCK v2 (Gootenberg et al., 2018)	Combination of RPA, T7 transcription, and Cas13 (Csm6) for amplifying the target gene and activating cis and trans cleavage, thus releasing the fluorescent signals.	Limited by the number of Cas proteins; Cross-cleavage between the protein and the reporter gene may influence the results.	Four different types of viruses or mutations can be detected simultaneously; Signal sensitivity increases by 3.5 times; Multiple diseases can be detected.
HUDSON+SHERLOCK (Myhrvold et al., 2018)	The combination of HUDSON, RPA, T7 transcription, and Cas13 for amplifying the target gene and activating cis and trans cleavage, thus releasing the fluorescent signals.	Simplify the nucleic acid extraction steps for only heating to eliminate nucleases but no special extraction or purification.	Rapid development and deployment in the sudden large-scale virus outbreak.
CARVER (Freije et al., 2019)	Combination of the antiviral activity of Cas13 and the diagnostic ability.	The Cas13 enzyme reduces viral RNA levels in cultured cells up to 40 times.	Application of both diagnosis and treatment of RNA viruses.
SHINE (Arizti-Sanz et al., 2020)	Single-step combination of RPA, T7 transcription, and Cas13 for amplifying the target gene and activating cis and trans cleavage, thus releasing the fluorescent signals.	Use an app to automatically analyze, interpret and report results to users.	Detection of COVID-19 out of hospitals and laboratories.
CARMEN (Katzmeier et al., 2019)	The combination of PCR/RPA and Cas13 for amplifying the target gene and activating cis and trans cleavage, thus releasing fluorescent signals.	CARMEN-Cas13 simultaneously detects hundreds of viruses.	Wide application to distinguish virus sequences at the species, strain, and single nucleotide polymorphism (SNP) level; The combination of CARMEN and NGS for pathogen detection, discovery and evolution.

### Detection of amplification products

Multiple CRISPR-Cas13-based methods with varying degrees of complexity are presently available for detecting amplification products. The most commonly used detection methods in the context of pathogenic microorganisms and based on isothermal amplification of nucleic acids include fluorescence methods, lateral flow assays (LFA), droplet-digital Cas13a assays (ddCas13a), colorimetric analyses, and microarray analyses (Table 4).

**Table 4:** Advantages and disadvantages of amplification product detection methods.

Detection	Advantage	Disadvantage
qPCR	High specificity	Can only detect high viral loads, so cannot be used immediately after initial infection; possibility of false negative results.
Fluorescence methods (East-Seletsky et al., 2016); Gootenberg et al., 2018	High specificity; high sensitivity; quantitative detection; simultaneous detection of multiple samples.	Requirement for a special detection instrument to capture fluorescent signals.
LFA (Maffert et al., 2017)	Easy to carry and store; low cost; simple operation; rapid detection; results are visible to the naked eye.	Qualitative results only; not amenable to high throughput.
ddCas13a (Granados-Riveron and Aquino-Jarquin, 2021, Tian et al., 2021)	High sensitivity; high specificity; absolute quantitative detection; wide applicability.	Not amenable to multiplex detection; requirement for special instrument to detect droplets.
Colorimetric analysis (Wang et al., 2016)	Simple and rapid detection; results are visible to the naked eye.	Can be difficult to distinguish color changes; lack of color identification standards; potential reading errors.
Microarray analysis (Ackerman et al., 2020)	High sensitivity and rapid detection; simultaneous detection of hundreds of samples.	Requirement for special instrument; results must be interpreted by trained technicians.

#### Fluorescence methods

In 2016, East-Seletsky et al. (2016) developed a novel CRISPR-Cas13a-based method for detecting target RNAs by introducing RNA fluorescent reporter molecules that are connected to both fluorescent and quenching groups. The activated Cas13a not only specifically cleaves the target RNA, but it also cleaves the RNA fluorescent reporter molecules away from the quencher. The fluorescent signal is thus released, and the fluorescent signal can be quantified. It has been reported that a detectable fluorescent signal can be produced during the 30 min detection of target RNA ranging from 1 to 10 pM (East-Seletsky et al., 2016). Featuring a lower background and higher signal-to-noise ratio, the fluorescent signal data can be read by a fluorometer, smartphone (Fozouni et al., 2021), LED light (Wang et al., 2020), or other simple devices. Currently, this novel method is able to detect ZIKV (Gootenberg et al., 2017), DENV (Gootenberg et al., 2017), Ebola Virus (Qin et al., 2019), PRRSV (Chang et al., 2020), BK polyomavirus (Kaminski et al., 2020), cytomegalovirus (Kaminski et al., 2020), and COVID-19 (Katzmeier et al., 2019).

#### Droplet-digital (dd) Cas13a

In the droplet-digital ddCas13a assay, the target RNA, Cas13a and oil are emulsified to produce thousands of pL-sized droplets. After recognition of a single target RNA by crRNA, the activated Cas13 protein collaterally cleaves more than 104 quenched fluorescent RNA reporter groups, and the released signal is sufficient to cause positive droplets to become fluorescent (Aquino-Jarquin, 2021; Tian et al., 2021; Figure 3C). Therefore, the ddCas13a assay allows the absolute digital quantification of a single unlabeled RNA molecule without the need for reverse transcription or amplification (Aquino-Jarquin, 2021; Tian et al., 2021). It has not only been applied to the accurate quantification of cell-free microRNAs in serum samples, but also to ultra-sensitive detection of 16S rRNA in urinary pathogens and accurate diagnosis of COVID-19 without the need for reverse transcription or sequence amplification. Due to the high sensitivity, single-molecule quantification, and wide applicability, ddCas13 has become a promising diagnostic tool (Tian et al., 2021). However, ddCas13a cannot be multiplexed and requires a special instrument to interpret the results.

#### Microarray analysis

CARMEN-Cas13 (Katzmeier et al., 2019) is a technology based on microarrays (Figure 3D). PCR or RPA amplification products and a Cas13 detection mixture (Cas13 protein, crRNA sequence, and reporter group) serve as the input for CARMEN-Cas13. These samples are prepared in a conventional microtiter plate and mixed with the unique, solution-based fluorescent color code. Each color-coded solution is emulsified in fluorine-containing oil to produce 1 nl droplets. Emulsified droplets from all of the sample and detection mixtures are pooled into a pipette and transferred to the microarray, in which two droplets are added in each well to pair all the droplets. Each well is physically separated on the glass substrate. The content of each well is determined by the color code of the droplet using a fluorescence microscope. The pair of droplets in each well fuses after exposure to the electric field, and then all detection reactions are initiated and monitored by a fluorescence microscope. CARMEN is able to diagnose dozens of samples simultaneously, and it can be quickly adapted to the detection of COVID-19 by incorporating an additional crRNA. CARMEN-Cas13 has also been verified to effectively identify several HIV drug-resistant mutations and subtypes of influenza A strains. The combination of CARMEN and NGS is promising in pathogenic detection; however, results should be interpreted by a well-trained staff in clinical nursing applications.

#### LFA

LFA is a popular method for diagnostic detection (Figure 3E). It uses cheap, light, and easy-to-store test paper, which can be stably stored for at least 1 year (Aquino-Jarquin, 2021). The test strips are usually composed of nitrocellulose membranes with a strong affinity for proteins. The test paper used in LFA does not influence the detection, and it is suitable for untrained users (Tian et al. 2021). Therefore, LFA is one of the most widely applied commercial diagnostic methods.

LFA is a paper-based platform that is based on antigen–antibody interactions or hybridization of single-stranded DNA targets. After mixing the sample with gold particles (AuNPs) conjugated to anti-6-carboxy-fluorescein (FAM) antibodies, a complex with FAM, RNA, and biotin bound to the AuNP-FAM antibody conjugation moves from the area of the sample application based on the capillary principle (Wang et al., 2016). If there is no target gene in the sample, Cas13 cannot be activated. During the lateral flow of the complex, biotin is blocked by the biotin ligand, and the control band detection area indicates a negative result. If there are target genes in sample, Cas13 is activated to cleave the RNA in the complex, thus separating FAM and biotin. The biotin ligand does not restrict the lateral flow of the complex, but it is blocked by the anti-FAM antibody, allowing visualizing of the test band, indicating a positive result (Wang et al., 2016).

The SHERLOCK-LFA test sensitively detects ZIKV and DENV ssRNA within 90 min at 2 aM (Safari et al., 2021). A recent study reported that the sensitivity of the fluorescence readout and lateral flow readout in the SHERLOCK-LFA test in 154 clinical samples of COVID-19 was 96 and 88%, respectively, and the specificity of both was 100% (Aquino-Jarquin, 2021). It has also been applied to detect Ebola and Lassa viruses (Maffert et al., 2017).

#### Colorimetric analysis

Colorimetric analyses monitor color changes of test solutions. These tests can be performed by the naked eye or with a colorimeter without the need for an expensive instrument. Therefore, it is a rapid, convenient assay (Wang et al., 2016). Yuan et al. (2020) designed and developed a colorimetric analysis platform based on CRISPR-Cas13a and AuNPs that can detect the African swine fever virus (ASFV) by the naked eye within 1 h. Based on the collateral cleavage activity of Cas13a/crRNA and Cas12a/crRNA, a linker ssDNA or RNA is designed to hybridize with AuNPs-DNA probes. In the absence of target RNA, the Cas13 protein is not activated, and the intact ssRNA in the reaction hybridizes with the AuNP-DNA probe. Then, the cross-linking of AuNPs induces an aggregation state, which changes the color of the colloidal solution from red to purple (Yuan et al., 2020). The activated Cas13a/crRNA recognizes the target RNA, which degrades ssRNA and depolymerizes AuNPs, thus maintaining the original color of colloidal solution. Despite the simplicity of this system, interpretation of the results of colorimetric analyses by the naked eye may be associated with errors due to a low capacity to distinguish color changes and a lack of a standard for color identification (Figure 3).

The optimal on-site rapid detection should be simply operated without expensive instrument, or it may even have no need for instrumentation, and the results should be easily interpreted by users. Therefore, colorimetric and FLA assays combined with isothermal amplification are promising for the rapid on-site detection of nucleic acids (Maffert et al., 2017). Fluorescence methods and microarray analyses are preferred to multiplex rapid detection, which can only qualitatively determine the absence or presence of pathogenic microorganisms (Huang et al., 2020; van Doremalen et al., 2020). In addition, nucleic acid samples are usually amplified before CRISPR-Cas13a processing, and the amplification steps sometimes may obscure or distort the true concentration of the original sample, leading to misleading results (Kebschull and Zador, 2015; Sabina, 2015).

## Summary and prospects

CRISPR-Cas diagnostic technologies are rapid and highly specific and can be deployed at a relatively low cost. Nevertheless, commercial CRISPR-Cas diagnostic kits or instruments are rare due to the complexity of translating emerging technologies into clinical practice. At present, relevant research on CRISPR-Cas diagnostic technology has been limited to laboratory experiments. With the massive global outbreak of COVID-19, it became apparent that many low-income and middle-income countries are unable to manufacture their own diagnostic test instruments, and importing them is expensive and takes precious time (Anwar et al., 2020). Therefore, rapid nucleic acid detection platforms with simple procedures and low costs are urgently needed to fight COVID-19 as well as future pandemics.

The present review summarizes the CRISPR-Cas-based methods for the detection of pathogenic microorganisms. Solving the noted problems that limit the three key steps in nucleic acid amplification and detection would permit optimization of current technologies. Thus far, a growing number of CRISPR-Cas13-based diagnostic tools have been developed. The key advantage of this technology is the applicability of less complicated instrumentation. These techniques are thus particularly suitable for application to epidemic outbreaks in resource-limited areas. However, the low throughput may result in low efficacy in detecting large-scale samples, such as clinical samples of COVID-19, and in cross-contamination, posing an important clinical challenge.

It is thus considered that a multidisciplinary combination of the CRISPR system, engineering, microelectronics, and miniaturization may increase detection throughput and automation. For example, isothermal amplification is a breakthrough relative to conventional PCR that has enhanced flexibility and has provided a novel selection of techniques for on-site detection. As research progresses, the combination of LAMP/RPA with CRISPR-Cas13 is expected to be even more widely applied to the rapid detection of pathogenic microorganisms. Moreover, the combination of isothermal amplification and the latest CRISPR Cas13 collateral cleavage technology significantly enhances detection accuracy and speed. Currently, great efforts have been made to further promote the combination of isothermal amplification and collateral cleavage technologies to reduce the cost and simplify procedures, aiming to optimize the on-site rapid detection of pathogenic microorganisms.

## Author contributions

ZH: methodology, investigation, project administration, writing—original draft, and data curation. JF: formal analysis, investigation, data curation, and visualization. MZ: data curation, visualization, validation, investigation, and resources. ZG: conceptualization, and supervision, and writing—review and editing. TX: conceptualization, writing—review and editing, and funding acquisition. All authors contributed to the article and approved the submitted version.

## Conflict of interest

The authors declare that the research was conducted in the absence of any commercial or financial relationships that could be construed as a potential conflict of interest.

## Publisher’s note

All claims expressed in this article are solely those of the authors and do not necessarily represent those of their affiliated organizations, or those of the publisher, the editors and the reviewers. Any product that may be evaluated in this article, or claim that may be made by its manufacturer, is not guaranteed or endorsed by the publisher.

## Footnotes

^1^ https://covid19.who.int/
